# Bone Matrix Levels of Dickkopf and Sclerostin are Positively Correlated with Bone Mass and Strength in Postmenopausal Osteoporosis

**DOI:** 10.3390/ijms20122896

**Published:** 2019-06-14

**Authors:** Thor Ueland, Lis Stilgren, Jens Bollerslev

**Affiliations:** 1 Research Institute for of Internal Medicine, Faculty of Medicine, University of Oslo, 0027 Oslo, Norway; 2 KG Jebsen TREC, University of Tromsø, 9010 Tromsø, Norway; 3 Department of Endocrinology, Svendborg Hospital, 5700 Svendborg, Denmark; lis.Stilgren@rsyd.dk; 4 Section of Specialized Endocrinology, Oslo University Hospital; Faculty of Medicine, University of Oslo, 0027 Oslo, Norway; jens.bollerslev@medisin.uio.no

**Keywords:** Wnt signaling, postmenopausal osteoporosis, bone matrix, bone mass, Dickkopf-1, sclerostin

## Abstract

Wnt signaling plays a pivotal role in maintaining bone mass. Secreted pathway modulators such as sclerostin (SOST) and Dickkopfs (DKKs) may influence bone mass inhibiting the canonical Wnt pathway. We evaluated whether bone protein content of secreted Wnt antagonists is related to age, bone mass, and strength in postmenopausal osteoporosis. We measured cortical and trabecular bone contents of SOST and Dickkopf-1 (DKK1) in combined extracts obtained after ethylenediaminetetraacetic acid and guanidine hydrochloride extraction in 56 postmenopausal women aged 47–74 (mean, 63) yr with a previous distal forearm fracture and a hip or spine Z-score less than 0. Our findings were (i) SOST and DKK1 protein levels were higher in trabecular bone, (ii) cortical and trabecular DKK1 and trabecular SOST correlated positively with bone matrix levels of osteocalcin (*r* between 0.28 and 0.45, *p* < 0.05), (iii) cortical DKK1 correlated with lumbar spine bone mineral density (BMD) (*r* = 0.32, *p* < 0.05) and femoral neck BMD (*r* = 0.41, *p* < 0.01), and (iv) cortical DKK1 and SOST correlated with apparent bone volumetric density and compressive strength (*r* between 0.34 and 0.51, *p* < 0.01). In conclusion, cortical bone matrix levels of DKK1 and SOST were positively correlated with bone mass and bone strength in postmenopausal osteoporotic women.

## 1. Introduction

Following menopause, bone remodeling accelerates and the amount of bone resorbed exceeds that subsequently formed by osteoblasts during bone formation. This imbalance underlies the increased bone loss in postmenopausal osteoporosis, a prevalent skeletal disorder characterized by low bone mass, compromised bone strength, and consequent increased risk of fractures [1]. A number of signaling pathways have been implicated in this age-related bone loss including a pivotal role for the wingless (Wnt) signaling pathway [2]. Activation of canonical Wnt signaling stimulates the generation of osteoblasts and production of bone while prolonging their lifespan [3,4], but also attenuates osteoclast differentiation and function by stimulating production of osteoprotegerin [5]. The Wnt pathways consist of a number of endogenous secreted pathway modulators such as sclerostin (SOST) and Dickkopfs (DKKs) that regulate bone mass by binding to lipoprotein receptor-related protein (LRP) 5 and LRP6 to inhibit the canonical pathway [6,7].

Thus, estrogen deficiency is associated with increased circulating SOST and Dickkopf-1 (DKK1) [8,9,10]. However, the association between systemic levels of these antagonists, bone mass, and strength are unclear. Low-circulating SOST was associated with low bone mass and osteoporotic fractures in several studies [11,12], but correlated positively with hip bone mineral density (BMD) in another study [13]. Similarly, evaluation of mRNA levels in bone suggested higher SOST and DKK1 in relation to fragility fractures [14], while another study demonstrated lower SOST and DKK1 mRNA in bone from postmenopausal women [15], positively correlated with BMD [16]. A positive association between DKK1 and SOST mRNA with BMD and lower fracture risk is at odds with the known function of these antagonists and suggests that bone matrix expression may reflect a different aspect of these antagonists. Furthermore, a complete demineralization and extraction of bone is not achieved using normal mRNA extraction protocols, which, for protein extraction, require 48 h using several demineralization and denaturing buffers.

The aim of this study was to evaluate whether bone protein content of secreted Wnt antagonists are related to age, bone mass, and strength in postmenopausal osteoporosis. This was achieved by extracting protein levels of the antagonists from transiliac biopsies, divided into cortical and trabecular compartments, from 56 postmenopausal women and correlating with BMD as well as ex vivo trabecular volumetric density and bone strength. 

## 2. Results

The demographic and baseline features of the study population are presented in Table 1. Women with postmenopausal osteoporosis were characterized by low bone mass and enhanced bone turnover, as determined by biochemical bone markers. 

**Figure 1:**
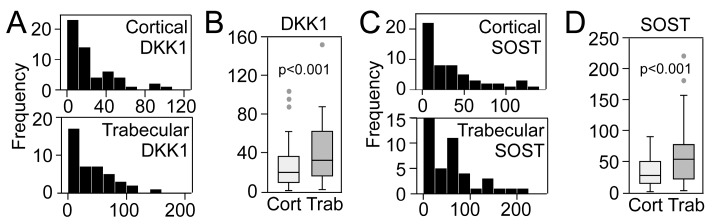
Distribution of cortical and trabecular bone matrix levels of (**A**) Dickkopf- 1 (DKK1) and (**C**) sclerostin (SOST) (ng/mg total protein) in 56 women with postmenopausal osteoporosis. (**B**,**D**) show comparison of trabecular and cortical levels of DKK1 and SOST, respectively.

We have previously reported extraction efficiencies for these Wnt antagonists showing that after the two extractions used in the study, specifically ammonium ethylenediaminetetraacetic acid (EDTA) and first guanidinium-HCl fraction, we were able to retain 93.4% and 98.7% for DKK-1 and SOST, respectively [17]. We were unable to detect Wnt inhibitory factor 1 (WIF1) and secreted frizzled-related protein 3 (sFRP3) in these samples. Figure 1A shows the left-skewed distribution of cortical and trabecular DKK1, normalized to total protein, in 56 transiliac crest biopsies from postmenopausal women, with markedly higher levels in trabecular bone (Figure 1B). A similar distribution (Figure 1C) with higher trabecular levels (Figure 1D) was observed for SOST.

### 2.1. Bone Matrix DKK1 and SOST and Association with Bone Mass and Strength

**Figure 2:**
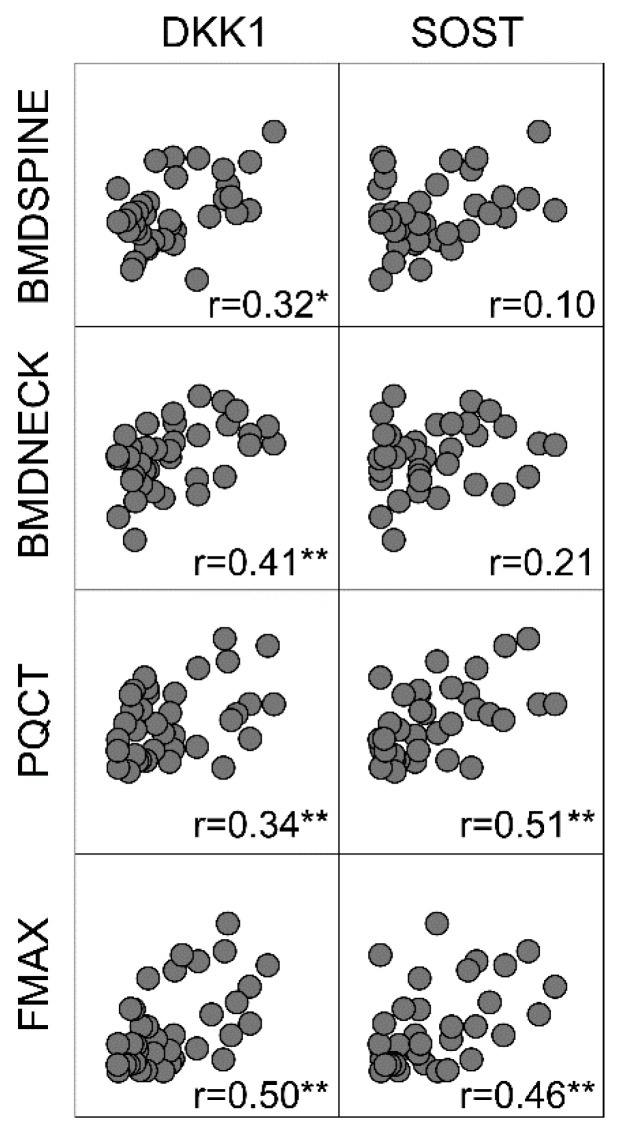
Correlation matrix (Spearman) of cortical bone contents of Dickkopf- 1 (DKK1) and sclerostin (SOST) (ng/mg total protein), bone mineral density (BMD) in the lumbar spine (*n* = 52), femoral neck (*n* = 50), and bone volumetric density (pQCT, *n* = 38), and biomechanical strength (Fmax, *n* = 37).

As shown in Figure 2 and the bottom of Table 2, lumbar spine and femoral neck BMD was positively correlated with cortical DKK1 and for spine, with trabecular DKK1. Finally, cortical DKK1 and SOST were positively correlated with bone volumetric density (pQCT) and biomechanical strength (Fmax).

### 2.2. Bone Matrix DKK1 and SOST and Correlation with Bone Turn–Over and Fractures

As shown in Table 2, no correlation between bone matrix levels of DKK1 or SOST and age or years since menopause was detected. Furthermore, no association with the calciotropic parathyroid hormone (PTH) or biochemical bone turnover markers carboxyterminal telopeptide of type I collagen (ICTP), serum alkaline phosphatase (sAP), or osteocalcin was detected. 

No association between bone matrix Wnt antagonist levels and bone calcium was detected. In contrast, cortical and trabecular DKK1 levels correlated positively with corresponding bone matrix osteocalcin levels. Trabecular SOST and osteocalcin were also positively correlated. 

Comparing Wnt antagonist levels in women with and without prevalent vertebral compression fractures revealed no difference for cortical DKK1 (*p* = 0.12) or SOST (*p* = 0.74), or trabecular DKK1 (*p* = 0.68) or SOST (*p* = 0.74).

## 3. Discussion

The main finding in our study was that cortical bone matrix contents of SOST and DKK1 in postmenopausal osteoporosis were positively associated with bone mass and strength. We were unable to detect sFRP3 and WIF1 in our bone specimens, suggesting a limited role of these Wnt antagonists in bone metabolism, or that they are not embedded in bone matrix during bone formation. However, DKK1 and SOST were readily quantifiable, and we were able to extract ~95% using the two extractions procedures utilized in our study. This is, to our knowledge, the first assessment of DKK1 and SOST protein levels in cortical and trabecular bone in postmenopausal osteoporosis and supports and extends previous findings by Jemtland et al. showing a positive correlation between mRNA levels of these antagonists and BMD [15]. 

Except for a correlation between trabecular DKK1 and spine BMD, the correlation between bone mass and Wnt antagonist levels was restricted to cortical bone in our study. However, a more prominent correlation between bone matrix DKK1, SOST, and osteocalcin was observed in trabecular bone. While circulating osteocalcin is used to reflect bone formation, the role of bone matrix osteocalcin is unclear and difficult to interpret [18]. Deletion of osteocalcin in experimental models increased cortical and trabecular properties [19], and in rats, this effect was restricted to trabecular bone [20]. Furthermore, bone fragments from osteocalcin knockout mice markedly reduced bone resorption in vivo [21], and conversely, osteocalcin fragments enhanced osteoclast function and activity in vitro [22]. Our previously reported negative correlation between osteocalcin levels in trabecular but not cortical bone and BMD in the current population may reflect a negative impact of osteocalcin on the skeleton [23]. With regard to Wnt signaling, Li et al., using transgenic β–catenin mice, demonstrated a higher expression and more prominent effect of alterations in Wnt/β–catenin signaling in trabecular, compared to cortical, bone [24]. Furthermore, transgenic overexpression of Wnt16 increased bone mass, with a stronger effect in trabecular bone [25]. With relevance to Wnt antagonism, overexpression and knockdown of secreted frizzled–related protein 4 (sFRP4) markedly reduced [26] and augmented [27] primarily trabecular bone mass. Taken together, alterations in bone matrix osteocalcin and canonical Wnt signaling seem to influence trabecular bone to a larger degree than cortical, and we speculate that the stronger association with osteocalcin in the trabecular compartment could reflect some kind of interaction, although the mechanism remains obscure. There are regional differences in pathophysiology, possibly related to higher bone turnover and increased skeletal blood flow in trabecular bone [28], making it more metabolically active and responsive to treatment [29]. 

Over–expression of DKK1 in mice shows that DKK1 markedly reduces osteoblast numbers and directly attenuates osteoblast matrix mineralization, resulting in osteopenia [30]. Conversely, deletion of a single allele of the *DKK1* gene increases osteoblast numbers and mineralization, leading to enhanced bone formation and bone mass [31]. These experimental data are supported by clinical studies in patients with multiple myeloma, where myeloma cells secrete DKK1 leading to lytic bone lesions by blocking osteoblast differentiation and enhancing receptor activator of nuclear factor–kappa–B ligand (RANKL)–dependent bone resorption in patients with high disease activity [32]. Similarly, sera from children with osteogenesis imperfecta contains elevated DKK1 and RANKL and inhibits osteoblast differentiation in vitro [33]. In patients with type 1 diabetes mellitus, high serum DKK1 and SOST were closely associated with both glycemic control and biochemical markers of bone turnover [34]. As for SOST, studies in human and mouse bone identify it as a secreted protein that may be transported to osteoblasts on the bone surface, where it may inhibit their differentiation and function [35]. Furthermore, sclerosteosis and Van Buchem disease, two rare bone disorders with mutations in or in close proximity of the SOST gene, leading to restricted expression of SOST, are characterized by progressive bone thickening due to enhanced bone formation [36,37]. Taken together, these studies in transgenic mouse models and clinical diseases characterized by dysregulated DKK1 and SOST expression show that these Wnt antagonists suppress osteoblast differentiation and function. 

**Figure 3:**
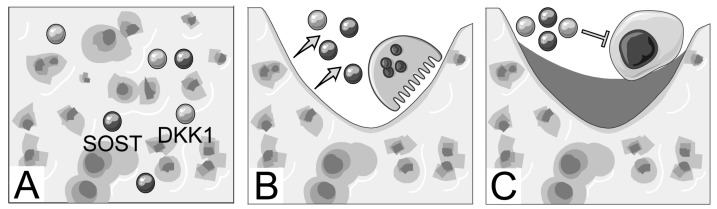
Proposed role of bone matrix Dickkopf–1 (DKK1) and sclerostin (SOST) in postmenopausal osteoporosis. (**A**) DKK1 and SOST are produced by osteoblasts or osteocytes during active canonical wingless (Wnt) signaling and stored in bone. (**B**) during bone resorption these are released and during excessive resorption they may (**C**) inhibit osteoblasts to the extent that they cannot replace the resorption lacunae.

Due to the established attenuating effect of DKK1 and SOST on the canonical Wnt signaling and osteoblast function, it seems unlikely that increasing bone matrix antagonist levels are actively inhibiting osteoblast function in the presence of higher BMD. The positive correlation between osteocalcin and DKK1 in cortical bone and with both antagonists in trabecular bone could suggest that the markers are incorporated in bone during bone formation. Indeed, DKK1 is directly regulated by the β–catenin/TCF complex under physiological circumstances, indicating that canonical Wnt activation during bone formation could increase DKK1 levels as a feedback loop [38]. While several growth factors have been hypothesized to be deposited in bone matrix during bone formation to act as delayed paracrine agents to couple bone remodeling [39], this role has not been described for DKK1 or SOST. In addition, osteocytes act as major regulators of bone remodeling by secreting factors that modulate the number and function of osteoblasts and osteoclasts [40,41]. Furthermore, osteocytes are embedded in bone matrix, are the main producers of sclerostin, and secrete considerable amounts of DKK1 [40]. We hypothesize that during the increased bone remodeling following menopause, DKK1 and SOST could be released from the bone matrix during bone resorption and contribute to an uncoupling by negatively regulating bone formation and promoting osteoclast formation and activity in a RANKL–dependent manner [42,43]. This unsynchronized remodeling balance would lead to decreased bone mass and, as bone formation slowed, the number of antagonists incorporated in bone would decrease correspondingly. This hypothesis is illustrated in Figure 3. 

Anabolic treatment modalities that enhance osteoblast–mediated osteoclastogenesis could have some negative side effects through such a mechanism. Indeed, daily injections with teriparatide in osteoporotic postmenopausal women, who have increased systemic DKK1, led to a further increase in DKK1 levels with a similar pattern as bone resorptive markers [10]. This was suggested to be compensatory due to augmented Wnt signaling or related to increased calcitriol production, which induces DKK1 in vitro [44]. Thus, combination therapy with anabolic treatment and anti–DKK1 or anti–SOST could potentially be beneficial by not only inhibiting DKK1 and SOST from active bone cells but also by inhibiting bone matrix–derived levels of these proteins released by enhanced osteoblast stimulated bone resorption. We speculate that release of DKK1 and SOST from bone matrix during increased bone resorption also could contribute to elevated systemic DKK1. We have previously shown that DKK1 may contribute to inflammation in vascular cells [45]; is a predictor of adverse outcomes in acute coronary syndrome [46]; and may have adverse metabolic effects [47], which could be relevant in postmenopausal women. Thus, high bone turnover or anabolic agents that lead to release of bone matrix–derived SOST or DKK1 could potentially have unfavorable cardio–metabolic effects. Indeed, intermittent teriparatide treatment has been shown to adversely affect glucose metabolism, inflammation, and endothelial function [48]. Obviously, this is conjecture, and it is unknown if bone matrix–derived DKK1 or SOST is functional and a significant source of systemic levels. Finally, biomarker analysis of bone biopsies is not a feasible way to predict osteoporosis and fracture risk in a clinical setting, and we do not propose that our findings can be utilized in risk prediction.

## 4. Materials and Methods 

### 4.1. Patients

Fifty–six postmenopausal women aged 47–74 years (mean, 63 yr), who had previously suffered a distal forearm fracture and had low BMD in the spine or hip (Z–score < 0), were included in the study as described previously [23]. From each patient, a transiliac crest bone biopsy was obtained under local anesthesia, using a modified Bordier trephine (inner diameter, 8 mm) from the standard site 2 cm below the iliac crest and 2 cm behind the anterior superior iliac spine. The samples were frozen at −80 °C immediately after removal. Still frozen, the biopsies were later sawed carefully to divide cortical and trabecular bone. None of the patients received treatment for osteoporosis when the iliac crest bone biopsy was performed. Written, informed consent for blood and bone biopsy collection was obtained from all patients. The study was conducted according to the Declaration of Helsinki II and the Guidelines of Good Clinical Practice and approved by the local ethical committee (1988/10302, 1 January 1989, The Regional Council in Central Denmark Region)

### 4.2. Serum Biochemistry

Blood samples were drawn after an overnight fast and either frozen at –80 °C until analysis or analyzed immediately. sAP was determined by spectrophotometry using p–nitrophenylphosphate as substrate [49]. PTH (1–84) was determined by radioimmunoassay (RIA) (Nichols Institute Diagnostics, Nijmegen, The Netherlands). sICTP was measured by an equilibrium RIA (Orion, Diagnostica, Turku, Finland). Serum osteocalcin was measured by an in–house RIA using rabbit antiserum against bovine gla–protein [50]. Intra– and interassay coefficients of variation were less than 10% for all assays.

### 4.3. Densitometry

BMD was determined by dual energy X–ray absorptiometry in the lumbar spine (L1–L4) and the left femoral neck using a QDR–1000/W scanner (Hologic, Inc., Waltham, MA, USA).

### 4.4. pQCT and Biomechanical Testing

pQCT was performed as previously described [51]. Briefly, each trabecular biopsy was placed in demineralized water in a plexiglass tube with a specially prepared cortical ring around the sample. Measurements were performed with a pQCT scanner (Stratec XCT 960 A, Stratec Medizintechnik, Pforzheim, Germany) in the center of the 5-mm long cylinder with a slice thickness of 1 mm (voxel size: 0.148 mm × 0.148 mm × 1 mm). The software supplied with the scanner was used for analysis of the data (version 5·10: center mode 1, peel mode 1, threshold 0·50, trabecular area 52%). The scanner was calibrated with a phantom each day before use. The center of the biopsy was found from the “scoutview”, as provided by the standard scanner software. After pQCT, the trabecular bone samples were tested in a material testing machine (Alwetron TCT5, Lorentzen & Wettre, Stockholm, Sweden) at a constant deformation rate of 2 mm min^−1^. During compression, force deformation curves were obtained, stored and subsequently analyzed by computer. Maximum force, Fmax (the force applied at the failure point), was determined directly from the force–deformation curves [51]. 

### 4.5. Preparation and Extraction of Bone Specimens

Bone samples were treated as described previously [52,53]. Briefly, cortical and trabecular bone specimens were washed with sterile water to remove soft tissue and blood, defatted in trichloroethylene for 6 days (changed every second day) at 4 °C, and dried by immersion in ethanol/ether (1:1). The samples were pulverized in a liquid nitrogen–cooled mortar and pestle, passed through an 84 mm sieve, and stored at −80 °C until use. For determining secreted Wnt antagonists and total protein contents, 15 mg of bone powder were extracted once with 1.5 mL of 0.5 mol/L ammonium EDTA (pH 6.2) and once with 1.5 mL of 4 M guanidinium–HCl (pH 7.4), both containing protease inhibitors. Extraction was carried out for 18 h at 4 °C by rotation; the solution was then centrifuged (12,000× *g* for 30 min) before the supernatant was separated from the remaining bone residues. Supernatants from both extractions were combined and desalted in Sephadex PD–10 columns (Amersham Biosciences, Piscataway, NJ, USA), lyophilized in a Speed Vac Concentrator (Savant Instruments, Hicksville, NY, USA), and stored at −80 °C until assayed. We have previously determined albumin in our bone samples, as a marker of blood contamination, and found no detectable levels [53]. Levels of osteocalcin were measured by RIA using a kit from DiaSorin, Inc. (Stillwater, MN, USA); calcium content was determined in extracts after HCl hydrolysis [52,53]; and total protein was measured spectrophotometrically, directly after desalting at 280 nm using BSA as a standard, as described by others [54]. These measures have been presented in these patients previously [23]. SOST, DKK1, sFRP3, WIF1, and total protein were determined in the extracts using matched antibodies from RnD Systems (Stillwater, MN, USA). 

### 4.6. Statistical Analysis

The distribution of DKK1 and SOST in matrix was skewed, and associations with other continuous measures were assessed using Spearman correlation. Wilcoxen signed–rank test was used to compare trabecular and cortical levels of Wnt antagonists. Two–sided *P*-values are presented and considered significant when <0.05. 

## 5. Conclusions

In conclusion, cortical bone matrix levels of DKK1 and SOST were positively correlated with bone mass and bone strength in postmenopausal osteoporotic women. Future studies are needed to elucidate whether DKK1 and SOST represent uncoupling factors and the clinical importance of these results.

## Figures and Tables

**Table 1:** Baseline characteristics of patients with postmenopausal osteoporosis.

Characteristic	Postmenopausal Osteoporosis	Normal Postmenopausal Range
Age in yr (mean, range)	63 (47–74)	
Years since menopause	16 ± 10	
sPTH (ng/L)	37 ± 14	30 ± 11
sICTP (μg/L)	3.2 ± 1.1	3.2 ± 1.0
sOCN (μg/mL)	16.6 ± 10.8	13.7 ± 7.1
sAP (U/L)	20 ± 5	11 ± 5
Prevalent vertebral compression fractures		
0/1/ > 1	49/4/3	
BMD (g/cm^2^)		
Lumbar spine, *n* = 52	0.71 ± 0.10	0.89 ± 0.11 ^†^
Femoral neck, *n* = 50	0.61 ± 0.07	0.73 ± 0.10 ^†^
pQCT (mg/cm^−3^), *n* = 38	198 ± 76	
Fmax (N), *n* = 37	67 ± 54	

*n* = 56 unless otherwise stated. ^†^ Standard hologic QDR1000 reference curves, age 60 yr. S, serum; PTH, parathyroid hormone; ICTP, carboxyterminal telopeptide of type I collagen; OCN, osteocalcin; BMD, bone mineral density; pQCT, apparent trabecular bone volumetric density; Fmax, compressive strength.

**Table 2:** Correlations (Spearman’s R) between cortical and trabecular contents of Dickkopf- 1 (DKK1) and sclerostin (SOST), bone turn-over, bone mass, and strength.

Characteristic	Cortical		Trabecular	
	DKK1	SOST	DKK1	SOST
Age	−0.13	−0.04	−0.19	−0.09
Years since menopause	−0.23	0.00	−0.30	−0.19
sPTH	−0.16	−0.20	−0.14	−0.04
sICTP	−0.10	0.07	0.00	0.09
sAP	0.22	0.03	0.20	0.08
sOCN	0.02	0.06	0.12	0.09
OCN ^†^	0.28 *	0.11	0.40 **	0.45 **
Calcium ^†^	0.20	0.13	−0.08	−0.09
BMD lumbar spine, *n* = 52	0.32 *	0.10	0.33 *	0.20
BMD femoral neck, *n* = 50	0.41 **	0.21	0.17	0.10
pQCT, *n* = 38	0.34 *	0.51 **	0.11	0.28
Fmax, *n* = 37	0.50 **	0.46 **	0.14	0.26

S, serum; PTH, parathyroid hormone; ICTP, carboxyterminal telopeptide of type I collagen; OCN, osteocalcin; BMD, bone mineral density; pQCT, apparent trabecular bone volumetric density; Fmax, compressive strength. * *p* < 0.05, ** *p* < 0.01. ^†^ Bone matrix levels.
